# The family as a determinant of stunting in children living in conditions of extreme poverty: a case-control study

**DOI:** 10.1186/1471-2458-4-57

**Published:** 2004-11-30

**Authors:** Hortensia Reyes, Ricardo Pérez-Cuevas, Araceli Sandoval, Raúl Castillo, José Ignacio Santos, Svetlana V Doubova, Gonzalo Gutiérrez

**Affiliations:** 1 Unidad de Investigación Epidemiológica y en Servicios de Salud, Centro Médico Nacional Siglo XXI, Instituto Mexicano del Seguro Social, México City, México; 2 Coordinación de Políticas de Salud, Dirección de Prestaciones Medicas, Instituto Mexicano del Seguro Social, México City, México; 3 Departamento de Epidemiología, Secretaría de Salud Estado de Guerrero, Chilpancingo, México; 4 Dirección General, Hospital Infantil de México "Federico Gómez", México City, México; 5 Unidad de Salud Pública. Dirección de Prestaciones Medicas, Instituto Mexicano del Seguro Social, México City, México

## Abstract

**Background:**

Malnutrition in children can be a consequence of unfavourable socioeconomic conditions. However, some families maintain adequate nutritional status in their children despite living in poverty. The aim of this study was to ascertain whether family-related factors are determinants of stunting in young Mexican children living in extreme poverty, and whether these factors differ between rural or urban contexts.

**Methods:**

A case-control study was conducted in one rural and one urban extreme poverty level areas in Mexico. Cases comprised stunted children aged between 6 and 23 months. Controls were well-nourished children. Independent variables were defined in five dimensions: family characteristics; family income; household allocation of resources and family organisation; social networks; and child health care. Information was collected from 108 cases and 139 controls in the rural area and from 198 cases and 211 controls in the urban area. Statistical analysis was carried out separately for each area; unconditional multiple logistic regression analyses were performed to obtain the best explanatory model for stunting.

**Results:**

In the rural area, a greater risk of stunting was associated with father's occupation as farmer and the presence of family networks for child care. The greatest protective effect was found in children cared for exclusively by their mothers. In the urban area, risk factors for stunting were father with unstable job, presence of small social networks, low rate of attendance to the Well Child Program activities, breast-feeding longer than six months, and two variables within the family characteristics dimension (longer duration of parents' union and migration from rural to urban area).

**Conclusions:**

This study suggests the influence of the family on the nutritional status of children under two years of age living in extreme poverty areas. Factors associated with stunting were different in rural and urban communities.

Therefore, developing and implementing health programs to tackle malnutrition should take into account such differences that are consequence of the social, economic, and cultural contexts in which the family lives.

## Background

Despite improvements in the health of children under five years of age, malnutrition remains as an important public health problem in Mexico [1] and in other developing countries [2-7]. Malnutrition in young children affects linear and brain growth and intelligence quotient, and is synergistically associated with child morbidity and mortality [8-13].

Previous reports have stated that malnutrition occurs mainly in rural areas and worsens under conditions of extreme poverty [4,14,15]. In Mexico, malnutrition is more frequent in the southern states, which are underdeveloped and have a predominantly indigenous population living in poor housing with unsanitary conditions [16]. The most recent nutritional survey at national level showed that the prevalence of stunting decreased during the last decade from 23% to less than 18%. Stunting is defined as the proportion of children under five years old whose height-for-age is less than -2 standard deviations of the reference population median. Wasting (the proportion of children under five years old whose weight-for-height is less than -2 standard deviations of the reference population median) has declined from 6% to 2%. However, analysis shows that prevalence of stunting is higher in rural (31.7%) than in urban strata (11.6%) [17]. The extent of malnutrition in extremely impoverished rural areas has fuelled implementation of public health programs aimed at improving children's nutritional status [18], but the impact of these programs has not been completely evaluated.

Poverty is not confined to rural areas, nor is malnutrition; they are also present in urban environments [19]. Uncontrolled and unplanned growth of cities has fostered the emergence of urban slums that lack basic sanitary services. In such settings, underprivileged families live overcrowded and malnutrition is undoubtedly present. Unfortunately, specific information about nutritional status of children living in urban slums cannot be obtained from existing epidemiological data. The extent of child malnutrition in these areas is probably underestimated because of a flawed health information system.

Malnutrition is an outcome of various factors resulting from unfavourable socioeconomic circumstances such as difficulties in obtaining food, unemployment -which determines an irregular income for the family's breadwinner-, limited access to education and health services, or illness caused by unsanitary conditions [2,5,14,20-24]. These circumstances are worsened by unequal access to, and distribution of resources among members of the family. However, some families are able to cope with such adverse environments and to maintain their children in an adequate nutritional status. The purpose of this study was to ascertain whether family-related variables constitute risk factors for stunting among young Mexican children living in extreme poverty, and whether these factors differ between rural and urban settings.

## Methods

### Study design

A case-control study was conducted from August through December 1998. Two extreme poverty level areas (one urban and one rural) were selected. Each area was analysed separately.

### Setting

The study took place in the south-western State of Guerrero, one of the poorest in Mexico, with a population of nearly three million.

The rural area, named Alto Balsas, is located next to the Balsas River; the urban area, named El Sinai, is a poor neighbourhood on the outskirts of the port of Acapulco, which has half a million inhabitants. For the rural area, four villages with a combined population of 4,638 were included in the study. The closest city is approximately 45 miles away. The principal language spoken in these villages is Náhuatl, and agriculture is the main economic activity. Because of seasonal conditions there is a high rate of cyclic migration each year. This migration occurs during harvest season, where farmers migrate from the State of Guerrero to another states. There is one medical doctor and one primary care centre per approximately 2300 people.

El Sinai is located in the outskirts of Acapulco and has 6,860 inhabitants, most people speak Spanish and they work in the local industry as unskilled manual workers. In this area, there is one medical doctor and one clinic per 3400 people. In both study areas, sanitation and public services are deficient. Table 1 shows the characteristics of both study areas.

**Table 1:** Characteristics of study areas

	**Alto Balsas^a^**	**El Sinai**
	**Rural area**	**Urban area**
Population	4,638	6,860
Distance to nearest urban centre	45–60 km	-----
Number of schools		
Kindergarten	4	2
Elementary school	4	2
Secondary school	0	1
Health Facilities (belonging to the Ministry of Health)	Rural clinic (2) Health post (2)	Urban clinic (1)
Health Personnel (employed by the Ministry of Health)		
Physicians	2	2
Nurses	3	2
Primary care technicians	2	2
Indigenous language	40–70%	4%^b^
Economic activities		
Agriculture	75–90%	0
Industry	5–20%	66%
Services	5–25%	12%
Annual migration^c^	30–50%	10%

^a ^Four communities

^b ^Estimations based on data from this study

^c^Proportion of families who migrate seasonally every year

### Study population

The units of study were children between 6 and 23 months of age. Children older than 6 months were included on the assumption that this is the minimum amount of time the child is exposed to family-related factors; also, this is the average age in which children are weaning (this is defined as the time when mothers begin to introduce food other than milk into the child's diet). Thus infants become more exposed to environmental causes of under-nutrition. The family was defined as next of kin, or persons sharing the same household and food expenses [25]. Only one child from each family was included in the study; if there was more than one eligible child, the oldest was selected. Infants with congenital diseases or those with low birth weights (less than 2,500 g) were excluded.

### Case definition

Cases were stunted children. This was ascertained by using the height-for-age indicator [26]. The criterion for stunting was: Z-value less than -2.00 standard deviations (SDs) below the median height-for-age [27].

### Control definition

Controls were children without stunting: equal to or above -2.00 SDs below the median in families that had no stunted children.

**The sample size **was calculated by using the case-control study formula in accordance with the following assumptions [28]: α = 0.05, β = 0.20, minimum risk to be detected = 2.5, proportion of controls exposed to the least frequent variable (migration) = 0.15, and case-control ratio 1:1. The required sample size was 112 children per group in each area.

### Study variables

A five-dimension framework to identify family-related factors that might have influence on the child's nutritional status was constructed. Each dimension comprises several variables selected to build up a comprehensive scenario.

The dimensions are the following:

1. Family characteristics: parents' age and literacy, type and duration of parents' union; family structure (nuclear or extended); presence of both parents (complete or incomplete family); number of members of the family and mother's use of contraceptives.

2. Family income: parents' employment (type of job, time on the same job, *per capita *family income), unemployment and migration during the past two years.

3. Household allocation of resources and family organisation: time spend by the mother to care for the child and to do domestic activities, and the way the family distributed and shared its income (percentage of income spent in food, clothes, transportation, rent, etc);

4. Social networks: type of networks (within or outside the family), size of networks, frequency of interactions and type of support (economic, child care, etc.).

5. Child health care: patterns of breast-feeding and health-care-seeking for preventive care (immunisations and Well Child Program visits) or curative care.

In addition, the following variables were included: child characteristics (age, sex, birth order and birth weight); and housing characteristics (type of construction, crowding conditions, indoor plumbing, sewerage system, and whether the kitchen was in a separate room).

### Data collection

In each study area, a local health worker able to communicate in Náhuatl and Spanish was trained to carry out a census to identify children fulfilling inclusion criteria, to interview the mother and to obtain the anthropometric measurements. Data were collected by using a pro-forma. The mother or caregiver was personally interviewed. During the visit, the interviewer measured and recorded the height and weight of each child aged between 6 and 23 months. The weight was measured using a digital scale with a precision error of ± 1 oz. The mother helped to measure the recumbent length of her child and this was done by using a portable calibrated board. To assure accuracy and reliability, one of the researchers (HR or RC) visited 10% of the households within the following week to confirm the data. There were no inconsistencies in data or anthropometric measures that could affect the results.

Mexican Institute of Social Security IRB and Ministry of Health authorities of the State of Guerrero approved the study. Local community leaders accepted and collaborated in the study and each family head as well as child's mother gave their informed consent.

### Statistical analysis

Analysis was carried out separately for each area. Firstly, a bivariate analysis was run; crude odds ratios (OR) and 95% confidence intervals (95%CI) were calculated for each variable in every dimension. The analysis included estimates of correlation and interaction among variables. Secondly, to obtain the best predictive model for stunting, all statistically or conceptually significant variables within each dimension were included in an unconditional multiple logistic regression analysis; the method selected for modelling started from a saturated model until finding the best explanatory model, after assessing the significance of each covariate and adjusting for major potential confounders such as age and sex of the child, literacy of the mother and household income. Once the best explanatory model was found, goodness of fit assessment was performed. The statistical analysis was carried out using SPSS (SPSS Professional Statistics 7.5 SPSS Inc. 1997) and STATA (STATA Statistical Software: Release 5.0 Stata Corporation, 1997).

## Results

Through the census, 326 eligible children aged between 6 and 23 months living in the rural area and 448 in the urban area were identified. Two hundred and forty-seven children (75.8%) from the rural area and 409 (91.3%) from the urban area who fulfilled the inclusion criteria were located. Because of the harvest season, some families living in the rural area migrated temporarily, so the remaining 24.2% of children could not be located.

Regarding the children living in the rural area 43.7% were stunted. Therefore, the group was divided into 108 cases and 139 controls; as to the urban area, 48.4% of children were stunted, resulting into 198 cases and 211 controls.

Table 2 shows children and housing characteristics of both study groups.

**Table 2:** Children and housing characteristics of study groups

**Variable**	**Rural**		**Urban**	
	
	**Cases n = 108 %**	**Controls n = 139 %**	**Cases n = 198 %**	**Controls n = 211 %**
**Child characteristics**				
Age in months (median, min-max)	16 (6–23)	13 (6–23)**	15 (6–23)|	12 (6–23)**
Sex (male)	53.7	53.2	58.1	48.4*
First birth order	11.1	20.9*	26.8	35.1*
Birth weight (g) mean (SD^+^)	2947(444)	2984(355)	3178(485)	3370(484)
**Housing characteristics**				
Dirt floor	87.0	85.6	40.9	31.1*
No indoor plumbing	78.7	71.9	60.6	49.3*
No sewerage system	100.0	100.0	39.4	28.0
No separate kitchen	87.0	79.9	57.1	44.1**
Overcrowding	79.6	64.7	48.0	33.2**

In the rural area, the proportion of first birth order children in the group of cases was lower than in the control group. Housing conditions were similar in both groups. Regarding urban children, cases had poorer housing conditions than controls; also, cases were older than controls in both study areas.

Table 3 shows the distribution of variables within each dimension.

**Table 3:** Distribution of variables by dimensions

**Variable**	**Rural**		**Urban**	
	
	**Cases n = 108 %**	**Controls n = 139 %**	**Cases n = 198 %**	**Controls n = 211 %**
**Dimension 1. Family characteristics**				
Mother's age (years)				
Median (min-max)	30 (18–43)	27 (15–44)	25 (15–43)	24 (16–47)
Father's age (years)				
Median (min-max)	32 (19–45)	30 (15–60)	28 (18–56)	28 (18–60)
Illiteracy of mother	50.0	37.4*	62.9	44.8**
Illiteracy of father	65.7	53.2*	50.3	46.2
Parents' civil status (married)	85.2	72.7**	63.1	68.2
Duration of parents' union longer than two years	95.4	84.2**	15.6	27.5**
Type of family (nuclear)	61.1	57.6	68.2	71.6
Completeness of the family	94.4	93.5	90.9	88.6
Size of the family (number of members) median (min-max)	8 (3–16)	7 (2–16)	5 (3–14)	5 (2–18)
Mother's use of contraceptive method	13.9	26.6*	58.7	69.6*
**Dimension 2. Family income**				
Mother's occupation				
Housewife	80.6	76.3	86.4	84.8
Other	19.4	23.7	13.6	15.2
Father's occupation				
Worker	13.0	20.9	27.8	20.4*
Farmer	65.721.3	53.2*	17.7	22.3
Other		25.9	55.5	57.3
Time during which father has worked in the same place (months) Median (min-max)	7 (1–30)	8 (0–36)	4 (1–60)	4 (1–30)
*Per capita *family income per month (USD)				
Mean (SD)	22.8 (11.7)	25.2 (6.0)	39.5 (18.6)	45.2 (24.1)*
migration of parents				
From rural to urban settings			71.2	55.8*
From rural to rural	2.8	8.6		
**Dimension 3. Household allocation of resources and family organisation**				
Child care provided exclusively by the mother	80.6	89.9*	89.9	87.2
Time spend by the mother to care for the child (hours/day) Median (min-max)	5 (3–17)	6 (0–21)	5 (0–21)	5 (1–14)
% of family income spent in:				
Food. Mean (SD^+^)	39.0 (13.5)	37.5 (14.9)	52.0 (15.6)	50.7 (14.8)
Transportation. Mean(SD^+^)	8.5 (5.9)	8.1 (4.1)	12.3 (7.3)	12.7 (7.0)
**Dimension 4. Social networks**				
Lack of social networks	24.1	24.5	14.1	14.7
Size of network (Small)	63.0	59.0	75.3	63.0**
Type of support				
Child care	64.8	50.7*	62.1	64.5
Economic	62.0	63.3	22.2	14.2**
**Dimension 5. Child health care**				
Breast feeding (months)				
Median (min-max)	7 (0–12)	6 (0–17)	4 (2–15)	3 (1–12)
Age at weaning (months)				
Median (min-max)	7 (1–14)	6 (1–13)**	4 (2–15)	4 (1–12)
Complete immunisation scheme	83.3	87.8	77.3	82.5
Attendance to the Well Child Program activities (number of visits)				
Median (min-max)	2 (0–4)	2 (0–9)	2 (0–6)	3 (0–8)**

* p < 0.05, **p < 0.01 (between cases and controls within the same area)

^+ ^Standard deviation

In the rural area, the following variables showed statistically significant differences when comparing cases and controls: parents' illiteracy, parents' civil status, duration of parental union, mother's use of contraceptive method, father engaged in farming activities, exclusive provision of care by the mother, social networks for child care, and age at weaning. At the urban area, mother's illiteracy, duration of parental union longer than two years, father's occupation, *per capita *family income, parents' migration from rural areas, size of social networks and frequency of attendance to the Well Child Program activities, were statistically significant variables.

Table 4 shows the results of the crude analysis of each dimension. In the rural area there was at least one significantly associated variable, while in the urban area there were two or more, except in dimension 3 (household allocation of resources and family organisation), in which no statistically significant variables were found.

**Table 4:** Variables associated with stunting, bivariate analysis

**Variable**	**Odds Ratio**	**Confidence interval (95%)**	**p value**
***RURAL AREA***			
**Dimension 1. Family characteristics**			
Duration of the parents' union:			
longer than two years	3.81	1.38 – 10.53	.01
two years or less	1.00		
Mother not using a contraceptive method	2.22	1.13 – 4.34	.02
Mother using contraceptive method	1.00		
**Dimension 2. Family income**			
Father occupation: Farmer	1.68	1.00 – 2.83	.04
Another type of job	1.00		
**Dimension 3. Household allocation of resources and family organisation**			
Child care: provided exclusively by mother	0.46	0.22 – 0.96	.03
shared with other caretakers	1.00		
**Dimension 4. Social networks**			
Family networks for child care	1.79	1.06 – 3.00	.02
Without family networks	1.00		
**Dimension 5. Child health care**			
Weaning: after six months of age	2.22	1.33–3.70	.002
at/before six months of age	1.00		
***URBAN AREA***			
**Dimension 1. Family characteristics**			
Illiteracy of mother	2.09	1.40 – 2.14	.001
Non-illiteracy of mother	1.00		
Duration of parents' union:			
longer than two years	2.04	1.24 – 3.35	.005
two years or less	1.00		
Family with more than 4 members	1.52	1.03 – 2.26	.03
Family with 4 members or less	1.00		
Mother not using a contraceptive method	1.61	1.06 – 2.42	.02
Mother using a contraceptive method	1.00		
**Dimension 2. Family income**			
*Per capita *family income:			
below $25 USD per month	1.65	1.03 – 2.64	.03
above $25 USD per month	1.00		
Father engaged at the same work place:			
equal or less than 2 years	1.84	1.19 – 2.82	.005
more than 2 years	1.00		
Parents migration:			
migrant from rural to urban area	1.96	1.28 – 2.99	.002
non migrant	1.00		
**Dimension 4. Social networks**			
Small networks	1.78	1.16 – 2.73	.008
Other size or without networks	1.00		
Family networks for economic support	1.72	1.03 – 2.87	.03
Without family networks	1.00		
**Dimension 5. Child health care**			
Number of visits to the Well Child Program:			
Less than two	2.43	1.58 – 3.74	.0001
Two or more	1.00		
Breast feeding: longer than six months	2.23	0.98 – 5.10	.05
six or less months	1.00		

Additionally, two of the child's characteristics showed significance (data not presented in the table): child's age (rural area, OR 4.5, CI95% 2.59 – 8.11; urban area, OR 1.98, CI95% 1.29 – 2.92), and child's sex (urban area, OR 1.45, CI95% 0.98 – 2.14; rural area not significant)

Table 5 presents the final explanatory models for each area after adjusting for some established risk factors (child's age and sex, maternal literacy, and household income). In the rural area, the model included only three variables belonging to dimensions two, three, and four. Dimension two (family income) showed an increased risk when the father's occupation was farmer. Dimension three (household allocation of resources and family organisation) showed that one covariate related to the mother's activities (the child being cared for exclusively by the mother) had a protective effect. Dimension four (social networks) showed that having family networks to provide care for the child entailed a higher risk.

**Table 5:** Variables associated with stunting, multivariate analysis*

**Variable**	**Odds Ratio**	**Confidence interval (95%)**	**p value**
RURAL AREA**			
Father's occupation: farmer	1.77	0.98 – 3.18	.05
Child care provided exclusively by the mother	0.30	0.13 – 0.69	.004
Family networks for child care	2.31	1.28 – 4.15	.005
URBAN AREA**			
Duration of parents' union longer than two years	1.89	1.01 – 3.54	.04
Parents migration from rural to urban area	1.57	0.95 – 2.59	.07
Father worked at the same place for 2 years or less	3.23	1.88 – 5.56	.0001
Small networks	2.11	1.27 – 3.49	.004
Less than 2 visits to the Well Child Program	2.57	1.54 – 4.30	.0001
Breast feeding longer than six months	1.71	0.62 – 4.73	.29

* Unconditional logistic regression analysis

**Adjusted by age and sex of the child, maternal literacy, and *per capita *family income in both areas

The explanatory model for the urban area included several covariates as risk factors in most dimensions. Dimension 1: migration of parents from rural to urban area, and duration of the parents' union longer than two years. Dimension 2: the father being in the same employment for two years or less. Dimension 4: having small family networks. Dimension 5: child having fewer than two visits to the Well Child Program activities during the past 6 months, and breast-feeding longer than six months. Age showed to be a significant confounder, and other characteristics of the child such as sex or birth weight, or those characteristics related to the parents such as literacy or *per capita *family income did not change the significance of the model when adjusted.

## Discussion

The role of the family as an important influence for nutritional status of children has been increasingly emphasized during the past few years, [29] and reinforced by the household production function perspective [30]. This is defined as "a dynamic process that occurs within the household to allow family members to combine their knowledge, resources and patterns of behaviour, either to promote, recover, or maintain health status" [31].

Nutritional status is an indicator of well being and malnutrition is the result of a complex process within which coexist a number of variables. The results in this study showed that the family related factors for stunting were different in each context -urban or rural-. The model highlighted the importance of identifying, [rather arbitrarily] a number of conceptually grounded dimensions that could be associated with stunting.

As mentioned earlier, rural and urban environments are different. Regarding the results of the rural area, within the family income dimension, the variable father's occupation (farming) was found to be a risk factor for child's stunting. In this area, availability of food depends upon local production, which in turn is related to father's occupation. Maize is the staple, and there is lack of food variability or inability to produce sufficient food for the family's nutritional requirements [32].

In the urban area the instability of the father's employment (working in the same place for less than 2 years) was found to be a risk factor. Lack of stable employment is a common problem among unskilled workers; their income is low and irregular, thus affecting their capacity to purchase goods and food, which in turn affects child nutrition [33]. Nevertheless, the other important variable included in this dimension, *per capita *family income, which was statistically significant in the bivariate analysis, did not show significance in the multivariate analysis. This finding could be interpreted as the ability of the family to cope with a difficult environment. Analysis of microeconomic variables shows the need of further studies to confirm plausibility of our findings and its association with malnutrition.

The dimension of family structure, which included socio-demographic characteristics of the parents, migration, and literacy attainment, has been repeatedly associated with the nutritional status of children. [2,5,20,23,34,35] In the urban area, some indicators of this dimension were associated with higher risk of malnutrition, particularly the longer duration of parents' union. It is possible that the economic and social burden on poor urban families with several children led the mother to give less attention to her younger children, whose nutritional status suffered in consequence. This result highlights the importance of considering the reproductive health sphere when developing health programs.

An interesting finding in this study was the relationship between social networks and children's nutritional status. It is often assumed that extended families have extensive social networks, and that this can be advantageous for the care of young children. However, we found quite the opposite in the rural area: the presence of family networks was associated with stunting, while exclusive provision of child-care by the mother showed a protective effect. These findings stress the role of the mother as primary caregiver, at least for young children; such findings also suggest that when a mother is present to care for the child, some of the effects of living in a poor community can be ameliorated.

In the urban area, conditions seem to be different, and the sizes of social networks influenced the nutritional status of the children positively. Urban families living in a more aggressive environment might be more likely to obtain health benefits if they have greater family or social support.

Use of health services, based on preventive measures from the Well Child Program, was also analyzed. Few visits to the Well Child Program activities were associated with stunting in the urban area. Restricted access to health care services in deprived areas is an important shortcoming with regard to monitoring child's growth and health [36]. Based on these findings, promotion of comprehensive services for children living in extreme poverty is relevant. Most health workers carry out preventive activities, among which nutritional status monitoring is essential. Therefore, it seems advisable to train these workers to recognise families showing some of the risk factors described in this study, so malnourished or at-risk children can be identified.

Migration was also explored. In the final explanatory model addressing the urban area, parents' migration from rural to urban area was found to be a risk factor for stunting. Migration from rural to urban settings is frequent among young people looking for improving their living conditions. However, families' adaptation to the new situation is a long process, given that such families live in a hostile environment and they have limited access to information regarding how to care for the child. In contrast, migration of one member of the family from one urban setting to another did not show a relationship with nutritional status of children. Adult male family members, mainly the heads of household, migrate to other states in Mexico or to the U.S. in search of better income. Perhaps the influence of this variable could be identified by a qualitative approach. Therefore, further research on this aspect is necessary.

The study has some limitations. Firstly, the study groups were of unequal size; this is a consequence of including all children aged from 6 to 23 months of age identified by the census. Additionally, since children of the control group were significantly younger than cases, some of them could have become stunted by the time they were of comparable age to the cases. To solve this limitation, the covariate age was adjusted in the multivariate analysis. However, it is possible that an age-matched cases-control design would have been preferable.

Secondly, the fact that almost 25% of eligible children in the rural area were not included due to migration of their families could have led to selection bias.

Another limitation is the lack of precise information regarding food practices among participating families. The interview allowed identifying general aspects such as duration of breast-feeding and age of weaning. Breast-feeding beyond 6 months of age entailed a risk of stunting, but only in the urban area. This issue has been controversial; some authors suggest that the relationship between prolonged breast-feeding and malnutrition may indicate a maternal decision to continue breast-feeding to a nutritionally disadvantaged child [37-39], rather than being the direct effect of prolonging breast-feeding on the child's nutritional status [40]. Further research in this area will contribute to the knowledge of cultural preferences about breast-feeding and its consequences on children's nutrition.

## Conclusions

This study suggests the influence of the family on the nutritional status of children under two years of age living in extreme poverty areas. Factors associated with stunting were different in rural and urban communities.

Therefore, developing and implementing health programs to tackle malnutrition should take into account such differences that are consequence of the social, economic, and cultural contexts in which the family lives.

## Competing interests

The author(s) declare that they have no competing interests.

## Authors' contributions

HR and AS conceived, designed and co-ordinated the study. RPC participated in the design of the study, statistical analysis and interpretation of data, and drafted the manuscript. RC co-ordinated the acquisition of data and participated in the statistical analysis. SD collaborated in the data analysis and drafted the manuscript. JIS and GG participated in the conception and design of the study, interpretation of data and critical revision for important intellectual content. All authors read and approved the final manuscript.

## Pre-publication history

The pre-publication history for this paper can be accessed here:


